# Promoting Equity in Parent Presence and Participation in Neonatal Intensive Care: Protocol for a Prospective Cohort Study

**DOI:** 10.2196/71930

**Published:** 2025-08-27

**Authors:** Marliese Dion Nist, Abigail B Shoben, Leif D Nelin, Lisa S Segre, Rita H Pickler

**Affiliations:** 1 Martha S. Pitzer Center for Women, Children and Youth College of Nursing The Ohio State University Columbus, OH United States; 2 Division of Biostatistics College of Public Health The Ohio State University Columbus, OH United States; 3 Division of Neonatology Nationwide Children's Hospital Columbus, OH United States; 4 Department of Pediatrics College of Medicine The Ohio State University Columbus, OH United States; 5 College of Nursing University of Iowa Iowa City, IA United States

**Keywords:** premature infant, neonatal intensive care units, parents, health promotion, health inequities

## Abstract

**Background:**

Parents of infants born preterm experience many barriers to their presence in the neonatal intensive care unit (NICU) and participation in infant caregiving. Parents from historically marginalized backgrounds or with limited social or economic resources may experience even more profound barriers, creating a significant source of health inequity for these parents and their infants. While the specific barriers and facilitators of parent presence and participation (PPP) in caregiving are unknown, PPP may be critical for improving clinical outcomes and neurodevelopment for infants born preterm.

**Objective:**

This study aims to (1) longitudinally determine the barriers and facilitators affecting PPP specific to parents with diverse sociodemographic characteristics, (2) determine the effect of PPP on infant clinical outcomes and neurodevelopment and the potential mediating effect of parent-infant responsiveness, and (3) determine the moderation effect of PPP on the relationship between infant stress exposure and infant clinical outcomes and neurodevelopment. We hypothesize that barriers and facilitators of PPP will vary based on sociodemographic characteristics and will change over the duration of a NICU hospitalization. We expect that higher levels of PPP will be associated with better infant outcomes.

**Methods:**

Parents (N=375) of infants born preterm, at <32 weeks’ gestational age, will be enrolled in a prospective cohort study. The parents will complete a daily survey documenting the amount of time spent in the NICU and the caregiving activities performed. The parents will also complete surveys at regular intervals during their infant’s admission to report barriers and facilitators of PPP, NICU-related stress, depressive symptoms, experiences of discrimination, and engagement with NICU staff. Additional data will be collected throughout each infant’s hospitalization, including invasive procedures, infant clinical data, and nursing documentation of PPP. The parents will complete the maternal-infant responsiveness instrument at the time of NICU discharge and at 3 months’ corrected age (CA). Infant clinical outcomes include the achievement of feeding milestones and the length of NICU stay. The infants will be assessed at 3 months’ CA using the test of infant motor performance and at 12 months’ CA using the Bayley scales of infant and toddler development, fourth edition.

**Results:**

Funding was awarded in August 2024. Data collection and analysis are expected to be completed by July 2029.

**Conclusions:**

By identifying the important barriers and facilitators of PPP over the duration of hospitalizations of infants born preterm and differences in these factors based on sociodemographic characteristics, time- and population-targeted interventions can be developed to remove system-level barriers and enhance facilitators. These efforts may increase PPP and promote health equity for diverse families.

**International Registered Report Identifier (IRRID):**

PRR1-10.2196/71930

## Introduction

### Background

Sociodemographic disparities exist in the outcomes of infants born very preterm (ie, infants born before 32 weeks’ gestational age [GA]) [1-4] and in the protective factors that promote more typical neurodevelopment. Parent presence in the neonatal intensive care unit (NICU) and participation in caregiving—protective factors associated with better clinical outcomes (eg, decreased length of stay) [5-8] and neurodevelopment [9]—are threatened by racial and socioeconomic disparities [10-13]. Although a few studies have reported lower parent presence and participation (PPP) for Black parents than for White parents [10-13] and for unmarried parents compared with married parents [11,12], the reasons underlying these disparities are unknown, and prior study findings are inconsistent, with some studies reporting associations between such factors and others reporting none [14]. Importantly, studies of PPP have been primarily conducted in the United States, followed by countries in Europe, with relatively few studies outside of these regions [14]. While factors associated with PPP have not been studied systematically, a combination of barriers and facilitators, many of which originate in the health care system, likely contribute to differences in PPP and the observed relationship with race and economic status. Our preliminary work, conducted as a survey study of NICU parents across the United States, revealed differences in the NICU experiences of diverse parents. Specifically, the most highly educated parents reported the highest levels of engagement with NICU staff; Black parents and Asian parents reported higher levels of discrimination compared with White parents [15]. However, whether these experiences affected PPP could not be determined from this cross-sectional study. A better understanding of barriers and facilitators and their relative influence on PPP will support the development of interventions to remove barriers and promote system-level changes that advance health equity for all infants born preterm and their parents.

PPP in infant caregiving (eg, feeding, diapering, bathing, holding, and skin-to-skin contact [SSC]) are necessary to ensure a timely, safe NICU discharge [16] and may also be important for improving infant outcomes [17,18]. PPP reduces the physical separation between parents and their hospitalized infants [19] and facilitates the development of responsive parent-infant relationships. Parent-infant responsiveness, defined as the prompt, contingent, and appropriate reactions that parents display in everyday parent-child interactions [20], develops through participation in early caregiving and is particularly important for the development of infant stress regulation [21] and positive cognitive outcomes [22,23]. PPP may also buffer the effects of unmodifiable influences on infant neurodevelopment, such as NICU stress exposure [24,25], which is associated with abnormal brain growth and neurodevelopmental impairment [26-28]. In the short term, there is some evidence that PPP buffers infant stress responses to painful procedures, resulting in lower pain scores and fewer behavioral distress signs compared with other nonpharmacological pain management interventions [29,30]. However, maternal skin-to-skin contact (SSC), the most commonly measured form of parent participation [17], while associated with lower mortality [31], better pain control [31], lower rates of hospital readmission [31], and more mature neurobehavior at NICU discharge [17], has unclear effects on length of stay [17] and longer-term neurodevelopment [31].

Despite the potential benefits of parent participation in caregiving, parent presence, a necessary precursor to participation in caregiving, is often unpredictable and infrequent [9,32], accounting for as little as 5% of the infant’s cumulative NICU hospitalization [9]. Moreover, although PPP may improve neurodevelopment and clinical outcomes for infants born preterm, infrequent parent presence is associated with more parent-reported emotional and behavioral problems during early childhood [33]. To develop interventions to promote PPP and potentially improve infant outcomes, the important barriers and facilitators and the specific factors affecting historically marginalized populations must be identified.

### Guiding Theoretical Model

**Figure 1:**
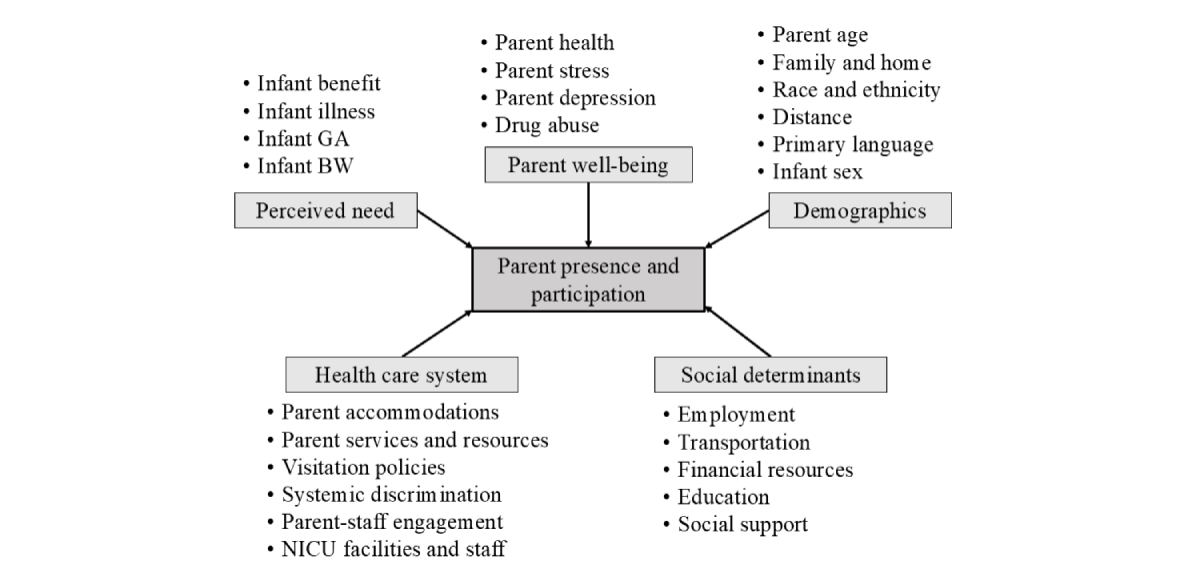
Barriers and facilitators of parent presence and participation. BW: birthweight; GA: gestational age; NICU: neonatal intensive care unit.

The *behavioral model of health services use* identifies the important constructs influencing engagement with the health care system, namely attributes of the health care system, the external environment (ie, social determinants), perceived need, health status, and demographic characteristics (Figure 1) [34]. The model has been previously used to guide qualitative research to determine the barriers and facilitators of parent SSC in the NICU [35]. Similar to SSC, PPP is likely influenced by factors within the health care system [10,32,35], social determinants that affect many health outcomes [12,13,36], the perceived need of parents to be close to their infant [35,37-39], the health status of parents (ie, parent well-being) [10,32,39], and demographic characteristics [10,36,37,39]. Aligned with these constructs from the model developed by Andersen, we have identified specific variables to be measured in this study based on a literature review and our prior research.

### Objectives

**Figure 2:**
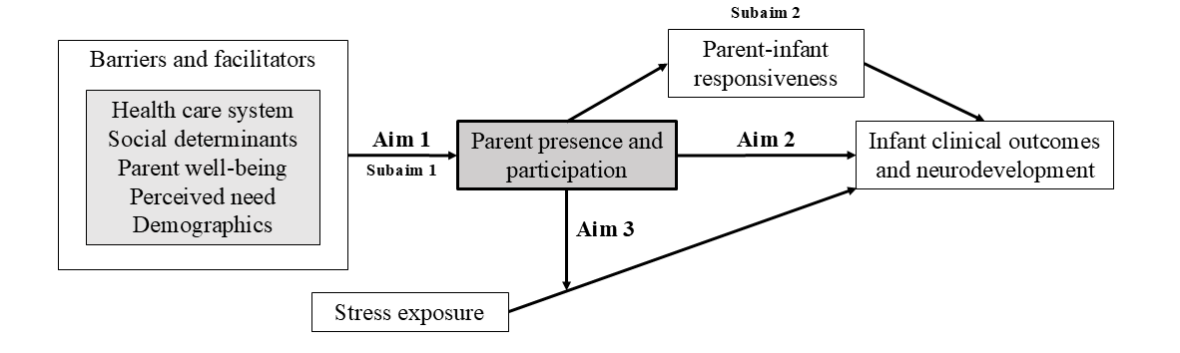
Conceptual model and aims.

Interventions are needed to eliminate barriers to PPP and reduce health inequities. However, foundational to this work is a better understanding of the barriers and facilitators of PPP and the effects on infant outcomes. This study aims to (1) longitudinally determine the barriers and facilitators affecting PPP specific to parents with diverse sociodemographic characteristics, (2) determine the effect of PPP on infant clinical outcomes and neurodevelopment and the potential mediating effect of parent-infant responsiveness, and (3) determine the moderation effect of PPP on the relationship between infant stress exposure and infant clinical outcomes and neurodevelopment (Figure 2). We hypothesize that the barriers and facilitators of PPP will be different for parents with different sociodemographic characteristics; parents with more influential barriers and fewer facilitators will have lower levels of PPP. We also expect that some of the barriers and facilitators—especially those related to the well-being of parents and the perceived need of parents—will change over time. Importantly, we expect that higher levels of PPP will be associated with more responsive relationships between infants and parents and, ultimately, better infant outcomes. Finally, we expect that PPP will attenuate the effect of NICU stress exposure on infant outcomes.

## Methods

### Study Design

We will use a nonexperimental, prospective observational design to address the aims. Study participation will begin within the first week of life for each infant and will continue until 12 months’ corrected age (CA; chronological age adjusted for degree of prematurity). Data will be used to build predictive models that include the most important barriers and facilitators of PPP for parents from different sociodemographic backgrounds.

### Participants and Setting

The primary participants in this study will be parents or primary caregivers with an infant born at <32 weeks GA and admitted to 1 of 3 NICUs in a large midwestern US metropolitan area. The NICUs include a level III 38-bed unit with primarily open bays and a few single-patient rooms; a level III 48-bed unit with single-patient rooms and a few double-occupancy rooms, which are typically reserved for multiple-birth siblings; and a level IV, all-referral, 48-bed unit with primarily single-patient rooms and a few beds in open bays (large room units typically with bedspaces separated only by curtains). Eligible parent participants will meet the following inclusion criteria: (1) be English- or Spanish-speaking, (2) be aged ≥18 years, and (3) have an infant born at <32 weeks’ GA who is expected to survive, based on assessment by the attending physician. Because approximately 20% of twin pregnancies and 65% of triplet pregnancies result in preterm birth before 34 weeks’ GA [40], parents of twins or higher-order multiples will be included. However, we will randomly select only 1 infant from a set of multiples to participate in the study.

The target sample size is 300 parents, which will ensure an adequate sample size to address the primary aim—barriers and facilitators of PPP. With 300 parents, we will be able to include up to 30 variables in multivariable models (assuming 5-10 observations per variable [41]), allowing for substantial flexibility in model selection. To account for attrition, we plan to enroll 375 parents. Assuming 2 parents enroll for 50% of infants and 1 parent enrolls for the remaining 50%, we expect to enroll 250 infants born preterm. To address disparities in PPP, we will enroll at least 150 Black or African American parents (40% of the sample). With an analytic sample of 120 Black or African American parents (after allowing for 20% attrition), we will be able to build an additional predictive model for PPP among Black or African American parents that could include 12 to 24 predictive and control variables.

### Measures

Data collection will begin at enrollment, within the first week of life for the infants, and will end when the infants reach 12 months’ CA. Data will be collected by parent report, the electronic health record (EHR), and NICU documentation.

### Barriers and Facilitators

To systematically identify barriers and facilitators related to the health care system, social determinants, perceived need, the well-being of parents, and demographics—and to examine the influence of these factors over time—we will collect data by parent report and from the EHR at regular intervals.

The parent-report of barriers and facilitators questionnaire (PRBFQ) is a 21-item, investigator-developed questionnaire that quantifies how often 11 potential barriers and 10 potential facilitators affect the ability of parents to be present in the NICU and participate in caregiving. The PRBFQ includes barriers, such as uncomfortable NICU facilities and lack of transportation, and facilitators, such as social support and employer-provided leave. It was pilot-tested and revised following a national survey study of NICU parents [15]. Parents will rate how often each barrier or facilitator affected their ability to be present and participate in caregiving using a 5-point Likert scale.

The discrimination in health care measure (DHCM) is a 7-item, self-report questionnaire that assesses the frequency of discrimination in interactions with health care providers and staff [42]. Across multiple studies of discrimination based on race, religion, sexual orientation and gender, and socioeconomic status, the DHCM demonstrated good internal consistency reliability [43]. The DHCM has been used to study perceived discrimination among White, Black, Hispanic, Asian, and Native American populations in various health care settings [43]. Perceived discrimination based on race, ethnicity, and socioeconomic status, as measured by the DHCM, is associated with poor patient-provider communication, health care mistrust, and lower satisfaction with the quality of health care [43]. Items are rated on a 5-point Likert scale. Summary scores are calculated as the mean of item ratings, ranging from 1 (no discrimination) to 5 (persistent discrimination) [43].

We will use the parent participation subscale of the empowerment of parents in the intensive care questionnaire to quantify parent-staff engagement in the NICU. In a sample of racially and socioeconomically diverse parents in the United States, the parent participation subscale demonstrated good internal consistency reliability (0.82) and was moderately correlated with measures of NICU satisfaction (ρ=0.39-0.46) [44]. The 8 items comprising this subscale are scored on a 5-point Likert scale. Summary scores are derived as the mean of item scores and range from 1 (no engagement) to 5 (high engagement).

The neonatal intervention score (NIS) will be used to measure infant illness severity, which may be related to the perceived need of parents to be close to their infant. Data to complete the 48-item NIS will be extracted from the EHR. The NIS has been validated in infants born preterm for use as a repeated measure to account for change based on normal physiological maturation or medical complications [45]. Psychometric testing in a sample of 99 infants born preterm revealed good internal consistency reliability (0.80) and validity [45]. The NIS scores in the first 24 hours of life were moderately correlated with other measures of illness severity. Furthermore, infants born preterm who did not survive to discharge had persistently higher median NIS scores over the course of hospitalization and higher peak NIS scores compared with survivors. Summary scores are calculated as a sum of weighted interventions, including the highest score for mutually exclusive items (eg, enteral tube feedings <75% and enteral tube feedings <100%); item weights range from 0 to 7. Infants receiving full oral feedings and no supplemental interventions receive the minimum summary score of 0. Because of the additive nature of the scale, there is no maximum summary score; summary scores in the validation study ranged from 0 to 32.5 [45].

The Patient-Reported Outcome Measurement Information System Short Form version 1.0–Depression 8b (PROMIS-D-8b) will be used to measure symptoms of depression in parents. The PROMIS-D short forms have demonstrated high internal consistency reliability across samples [46,47] and validity in males and females, as well as in racially, ethnically, and socioeconomically diverse populations [47]. The PROMIS-D short forms are also highly correlated with other measures of depression (ie, Edinburgh Postnatal Depression Scale, Center for Epidemiologic Studies Depression Scale, and Beck Depression Inventory); they are responsive to change over time and can be compared easily with these other measures [48,49]. The PROMIS-D-8b includes 8 items, rated on a 5-point Likert scale, with higher scores indicating more depressive symptoms. Summary scores are reported as standardized *t* scores with a mean of 50 and an SD of 10, based on the general US population [50].

In addition to depression, we will also measure NICU-related stress in parents using the parental stressor scale: NICU (PSS: NICU), a 20-item instrument with 3 subscales assessing parental perceived stress related to the NICU environment [51]. The internal consistency reliability coefficients of the overall PSS:NICU and its subscales are good [51]. Construct validity has been established, and the subscales are generally correlated [51]. Items are rated on a 5-point Likert scale. Summary scores are calculated for each subscale as a sum of item scores.

### Measurement of PPP

The parents will report their PPP using the parent participation diary. The parents will complete the diary daily, recording the duration of their presence in the NICU. If present on a given day, the parents will also report the number of minutes of SSC or holding and the number of diaper changes, bathing episodes, or feedings in which they participated; these will be converted to minutes of participation (ie, 5 min for a diaper change, 20 min for bathing, and 30 min for a feeding) based on previous research [52]. The parent-reported data will be verified using NICU sign-in logs and the nursing documentation in the EHR. The PPP will be operationalized as a duration (ie, time in min) and a frequency. For frequency, presence will be defined as the percentage of days during the infant’s hospitalization that the parents were present. Participation frequency will be defined as the percentage of days present that were spent providing infant care.

### Infant Outcomes

We will measure infant clinical outcomes, including the length of stay and the achievement of oral feeding competence, using data extracted from the EHR. The achievement of oral feeding competence is typically a criterion for NICU discharge [16]. Oral feeding skill is also an early indicator of neurobehavioral development and an important predictor for future neurodevelopmental outcomes [53]. We will measure the achievement of 3 indicators of feeding competence: time from the first oral feeding to full oral feedings, time from the first oral feeding to hospital discharge, and time from full oral feedings to hospital discharge. Each indicator will be measured in days, following published protocols [54,55]. Full oral feedings are achieved when the infant is consuming all nutrients orally (formula or breast milk) without intravenous or enteral tube supplementation, for 24 hours.

In addition to the clinical outcomes, we will measure infant neurodevelopment at 3 and 12 months’ CA, using the test of infant motor performance (TIMP) and the fourth edition of the Bayley scales of infant and toddler development, fourth edition (BSID-IV), respectively. The TIMP, a norm-referenced assessment of early motor performance, evaluates spontaneous movements and postural control in infants born preterm up to 4 months’ CA [56]. The TIMP has demonstrated excellent reliability [57,58] and validity [57] and is responsive to change over time [56,57]. In infants born very preterm, TIMP scores at 3 months’ CA are significantly associated with cognitive, language, and motor performance on the BSID at 2 years’ CA [59]. The test begins with an observation for 13 spontaneous movements, scored as present or not present, followed by 29 elicited items that measure head and trunk control and are scored from 4 to 7 points. The TIMP raw scores are converted to *z* scores, based on age-equivalent standards for CA at the time of assessment [56]. At 12 months’ CA, we will use the BSID-IV, a measure consisting of 5 scales of development: cognitive (81 items); language—receptive (42 items) and expressive (37 items); motor—fine (46 items) and gross (58 items); social-emotional (35 items); and adaptive behavior (10 skill areas). The number of items administered is based on the child’s age at the time of testing. Raw scores can be converted to scaled scores, which can then be converted to composite score equivalents. The internal consistency reliability of the scales ranges from 0.85 to 0.98 [60].

### Parent-Infant Responsiveness

We will use the maternal-infant responsiveness instrument (MIRI), a 22-item self-report scale that measures parent responsiveness to their infant, recognition of infant responsiveness to the parent, and difficulties in responsiveness [61]. Participants will respond using a 5-point Likert scale, with higher scores indicating higher responsiveness. A recent integrative review of 15 interdisciplinary studies found good internal consistency reliability in samples including both mothers and fathers [54,55] and both White and Black or African American parents [62]. The MIRI is highly inversely correlated with measures of stress, anxiety, and depression, and highly directly correlated with measures of self-efficacy and parental competence [62]. The MIRI is most commonly used when infants reach term-equivalent age (similar to the age at NICU discharge) and during the first 6 months’ CA [62]. Parents will complete the MIRI at discharge or 40 weeks’ postmenstrual age, whichever comes first, and at the time of the infant’s outpatient follow-up appointment at 3 months’ CA.

### Stress Exposure

We will use a count of invasive procedures to quantify stress exposure in the NICU. Researchers commonly quantify stress exposure as a count of skin-breaking procedures [26], which may include other noxious procedures, such as nasogastric tube insertion [63], endotracheal intubation [63,64], or tracheal suctioning [63], and have found associations with brain maturation and neurodevelopment [63,65]. Because procedures, such as nasal, oral, or tracheal suctioning [66], intubation [67], and enteral feeding tube insertion [68] are associated with stress responses in infants, we will measure stress exposure as a count of invasive procedures, defined as procedures or procedural attempts that break the skin or involve the insertion of a foreign object into the body.

### Procedure

Data collection will begin after written informed consent is obtained. The parents will complete surveys at regular intervals by either directly entering data into REDCap (Research Electronic Data Capture; Vanderbilt University) [69,70] hosted at The Ohio State University and accessed through a personalized, emailed link or using paper-and-pencil surveys, based on their preference (Table 1). Parent-reported NICU data collection will begin at the time of enrollment and will conclude when the infants reach 40 weeks’ postmenstrual age or are discharged from the NICU, whichever comes first. The number of surveys and their frequency will depend on the infant’s GA at birth and chronological age, respectively, with parents of the youngest infants, born at 22 weeks GA, completing surveys for up to 18 weeks, and parents of infants born at later GAs completing fewer. We will collect EHR data throughout each enrolled infant’s hospitalization to obtain clinical data needed to complete the NIS, feeding competence, and stress exposure measures. At discharge, infants born preterm at <32 weeks GA are scheduled for routine follow-up appointments in the developmental follow-up clinic. The parents will complete their final survey when their infant returns for the first developmental follow-up appointment at 3 months’ CA. We will extract data from the EHR for the TIMP and the BSID-IV after each enrolled infant’s follow-up appointments in the developmental clinic at 3 and 12 months’ CA, respectively.

**Table 1:** Parent-report survey data collection time points.

Measure	At enrollment	Collection intervals during the NICU^a^ stay	At discharge^b^	At 3 months’ CA^c^
Demographic form	✓	Once	—^d^	—
Participation diary	✓	Daily	—	—
PRBFQ^e^	✓	Every 2 weeks	—	—
DHCM^f^	✓	6 and 12 weeks of life^g^	—	—
EMPATHIC-PP^h^	✓	6 and 12 weeks of life^g^	—	—
PROMIS-D-8b^i^	✓	6 and 12 weeks of life^g^	—	—
PSS^j^: NICU	✓	6 and 12 weeks of life^g^	—	—
MIRI^k^	—	—	✓	✓

^a^NICU: neonatal intensive care unit.

^b^Data collected at discharge or 40 weeks’ postmenstrual age, whichever comes first.

^c^CA: corrected age.

^d^Not applicable.

^e^PRBFQ: parent-report of barriers and facilitators questionnaire.

^f^DHCM: discrimination in health care measure.

^g^Data collected at 6 weeks of life or discharge, whichever comes first; and at 12 weeks of life, 40 weeks’ postmenstrual age, or discharge, whichever comes first.

^h^EMPATHIC-PP: empowerment of parents in the intensive care–parent participation.

^i^PROMIS-D-8b: Patient-Reported Outcome Measurement Information System Short Form version 1.0–Depression 8b.

^j^PSS: parental stressor scale.

^k^MIRI: maternal-infant responsiveness instrument.

### Statistical Analysis Plan

To determine the barriers and facilitators affecting PPP and examine changes in these factors over time, we will use generalized estimating equations (GEEs) to estimate the effects of predictors on PPP as both a frequency and a duration. The GEE approach allows for clustering of observations from the same individual. We will conduct this analysis for the entire sample and for subgroups of participants, focusing on parent race and socioeconomic status (ie, low, moderate, and high), to address reported disparities in PPP. The predictors will be operationalized as summary scores from the DHCM, parent participation subscale of the empowerment of parents in the intensive care, PROMIS-D-8b, and PSS:NICU (as described earlier for each measure); each barrier or facilitator from the PRBFQ, categorized as not present (Likert=1), somewhat influential (Likert=2-3), or highly influential (Likert=4-5); and demographic characteristics. We will estimate the relative increase or decrease in PPP based on differences in levels of these factors in 2-week intervals [71] using a variable selection approach tailored for GEE [72] to identify the most important predictors of PPP. The resulting statistical models will include the most important predictors associated with PPP.

To determine the effect of PPP on infant clinical outcomes and neurodevelopment, we will use linear regression models, with each outcome (clinical or neurodevelopmental) measure modeled as a function of parent presence, total time providing SSC, and total time providing other care. We will estimate the effects of parent presence and all forms of parent participation, including the potentially unique effects of SSC. To determine the mediation effect of parent-infant responsiveness on the relationship between PPP and infant clinical outcomes and neurodevelopment, we will conduct standard mediation analyses using bias-corrected bootstrapped CIs. These analyses will be conducted using PROCESS (Andrew Hayes), a macro compatible with multiple statistical software programs, for inferential testing [73].

To determine the moderation effect of PPP on the relationship between infant stress exposure and infant clinical outcomes and neurodevelopment, we will use linear regression and model each infant’s outcome as a function of stress exposure (defined as a count of invasive procedures), parent participation (defined as total time in minutes providing care), and the interaction between stress exposure and parent participation.

### Ethical Considerations

The study was approved by the institutional review board at Nationwide Children’s Hospital (STUDY00004110) in April 2024. Parents will provide written informed consent for themselves; 1 parent is required to provide written informed consent for the infant’s inclusion. Participation is completely voluntary and will not affect clinical care. Parents may withdraw consent for themselves and their child at any time. Data confidentiality will be maintained throughout the study using standard best practices, including coding of data, secured storage spaces for data collected on paper forms, and password-protected, encrypted servers for data stored in electronic forms. The data repository that will be made available when the study is completed will be deidentified. Parents will receive compensation for completing all data surveys, including the daily diary. Compensation will be provided weekly, and the amount will depend on the number of surveys completed, with a maximum compensation of US $325 for parents of infants born at 22 weeks' GA, the youngest possible GA for the study.

## Results

Grant funding from the National Institutes of Health was awarded on August 15, 2024. As of January 2025, all surveys and data forms have been created and tested in REDCap. Participant recruitment began in February 2025; 66 parent participants and 43 infant participants have been enrolled as of June 30, 2025. Data collection began in March 2025 and is expected to be complete in February 2029 when all infants are >12 months’ CA. Data analysis will be performed after all data have been collected with expected results in the spring and summer of 2029.

## Discussion

### Anticipated Findings

The overarching objective of this study is to determine the barriers and facilitators associated with PPP and identify differences among racial and socioeconomic groups. This information is needed to develop interventions that promote health equity for parents and infants. Unfortunately, robust data on PPP are lacking. This may be because PPP is difficult to quantify, resulting in measures across studies that cannot be compared. Systematic reviews of PPP reporting data sources and measures used to quantify PPP have shown that nursing documentation is the most common method for collecting data on parent presence, followed by parent report and unit visitor logs; few studies use >1 data source [14]. Furthermore, the frequency of presence—measured as the proportion of days visited during hospitalization—is the most common measure of parent presence, followed by duration of presence. Few studies incorporate both measures [14]. Data on parent participation in NICU caregiving are lacking. Parent participation is most commonly conceptualized as SSC, with little inclusion of other critical caregiving activities (eg, feeding and bathing) [17]. This study will address these limitations by quantifying the frequency and duration of parent presence as well as the caregiving activities in which parents participate.

While many researchers have investigated the demographic predictors of PPP, such as the age of the parent, socioeconomic status, and race, or infant characteristics, such as the GA at birth, the length of the NICU stay, and the sex [14], this study focuses on the modifiable factors, with an emphasis on those originating from the health care system, that may disproportionately affect parents from racially underrepresented groups or those lacking financial and social resources. This is an important aspect of the study—the recognition that individual parents cannot be held responsible for the barriers originating within systems. Rather, it is the system that must respond to the needs of the parent and the infant and create environments and processes that encourage presence and participation. We are including demographic and clinical variables so that we can develop population-targeted interventions that may differ across groups of parents. To date, researchers have not prospectively determined the relative importance of specific barriers and facilitators of PPP or the changes in these factors over the period of hospitalization, thus limiting the ability to develop time- and population-targeted interventions. This will be the first study to systematically measure PPP, the barriers and facilitators of PPP, and the specific effects of PPP on infant outcomes. By using a theory-guided approach to understanding the barriers and facilitators of PPP, we will be prepared to develop interventions that equitably promote PPP and improve outcomes for infants born preterm and their families.

### Strengths and Limitations

The proposed study has several strengths. First, it centers on parent equity by seeking to identify the barriers to PPP in the NICU. Furthermore, the large sample size, longitudinal analysis, and comprehensive data collection will allow us to demonstrate the effect of PPP on both the child and the parent.

There are also limitations. We may need to extend recruitment beyond the initially identified sites. Fortunately, there are additional local NICUs within the identified hospital system that we can include without further institutional review board review. We may still encounter challenges in recruiting a diverse group of parent participants, which could also necessitate adding another NICU with greater diversity, even if it has fewer admissions. Although we intend to recruit a diverse sample of parents, our efforts may be constrained by the demographic characteristics of the accessible population. We expect to enroll 150 Black or African American parents, but we may be unable to recruit a sufficient number of parents from other races or ethnicities to facilitate meaningful analysis. Similarly, although all surveys have been professionally translated into Spanish, historical admissions data from the hospital system suggest that only a limited number of Spanish-speaking participants will be eligible. Thus, our analysis will be limited to including language as a possible predictor of PPP; we will not be able to build predictive models specifically for Spanish-speaking parents. We will also need to minimize missing data; therefore, we will maintain daily contact with parent participants. This will include daily “rounds” by a research team member in each NICU, during which we will greet parents if they are present or leave a short note for them at their infant’s bedside. We will also use SMS text message reminders for parents who choose this option, sending reminder SMS text messages and follow-up texts if surveys are not submitted. To reduce missing data for neurodevelopmental outcomes assessed during routine neonatal follow-up appointments, we will contact parents before their scheduled appointments at 3 and 12 months’ CA and will send birthday cards for infants on their 1-year birthday, which will fall between these appointments.

### Conclusions

Data from this study will be used to develop time- and population-specific interventions to remove the barriers and enhance the facilitators that affect PPP in the NICU. In doing so, we aim to improve infant outcomes and reduce disparities in PPP and infant outcomes, thereby promoting health equity for all infants born preterm and their parents.

## Appendix

Multimedia Appendix 1 Peer review report from ICSc - Interdisciplinary Clinical Care in Specialty Care Settings Study Section (National Institutes of Health, USA).

## Abbreviations

BSID-IV: Bayley scales of infant and toddler development, fourth edition
CA: corrected age
DHCM: discrimination in health care measure
EHR: electronic health record
GA: gestational age
GEE: generalized estimating equation
MIRI: maternal-infant responsiveness instrument
NICU: neonatal intensive care unit
NIS: neonatal intervention score
PPP: parent presence and participation
PRBFQ: parent-report of barriers and facilitators questionnaire
PROMIS-D-8b: Patient-Reported Outcome Measurement Information System Short Form version 1.0–Depression 8b
PSS: parental stressor scale
REDCap: Research Electronic Data Capture
SSC: skin-to-skin contact
TIMP: test of infant motor performance

## Data Availability

Data sharing is not applicable to this article as no datasets were generated or analyzed.
