# Supplementary material for: Promoting Equity in Parent Presence and Participation in Neonatal Intensive Care: Protocol for a Prospective Cohort Study
Source: JMIR Res Protoc. 2025 Aug 27;14:e71930. doi: 10.2196/71930 (PMC12423611; doi:10.2196/71930)
Supplement: Multimedia Appendix 1 [file resprot_v14i1e71930_app1.pdf]

SUMMARY STATEMENT

PROGRAM CONTACT:
MICHELE Walsh
301-402-7886
michele.walsh@nih.gov

(Privileged Communication)

Release Date: **02/24/2024**
Revised Date:

Application Number: **1R01HD113525-01A1**
Formerly: **1R01HD113525-01**

Principal Investigators (Listed Alphabetically):

NIST, MARLIESE D (Contact)
PICKLER, RITA H

Applicant Organization: OHIO STATE UNIVERSITY

Review Group: **ICSC**
Interdisciplinary Clinical Care in Specialty Care Settings Study Section

Meeting Date: **02/08/2024**
Council: **MAY 2024**
Requested Start: **07/01/2024**

Opportunity Number: **PA-20-185**
PCC: **PPB -MW**
Dual IC(s): **NR**

Project Title: **Equity for Parent Presence and Participation in Caregiving in the NICU**

SRG Action: **Impact Score:20 Percentile:6**
Next Steps: **Visit https://grants.nih.gov/grants/next_steps.htm**
Human Subjects: 30-Human subjects involved - Certified, no SRG concerns
Animal Subjects: 10-No live vertebrate animals involved for competing appl.
Gender: 1A-Both genders, scientifically acceptable
Minority: 1A-Minorities and non-minorities, scientifically acceptable
Age: 3A-No children included, scientifically acceptable

Project Year	Direct Costs Requested	Estimated Total Cost
1	[redacted]	[redacted]
2		
3		
4		
5		
TOTAL	[redacted]	[redacted]

ADMINISTRATIVE BUDGET NOTE: The budget shown is the requested budget and has not been adjusted to reflect any recommendations made by reviewers. If an award is planned, the costs will be calculated by Institute grants management staff based on the recommendations outlined below in the COMMITTEE BUDGET RECOMMENDATIONS section.

1R01HD113525-01A1 NIST, MARLIESE

RESUME AND SUMMARY OF DISCUSSION: This resubmitted application proposes to conduct a longitudinal cohort study to examine the association between parental presence and participation (PPP) in caregiving and infant outcomes over time in the neonatal intensive care unit (NICU). The review panel agreed that the proposed research is of high significance given the fact that it directly addresses an important knowledge gap for developing interventions targeting system-level changes for greater health equity for families. The rigor of the prior research is strong and supported by the teams' previous work. During the discussion, the panel noted that the study team of this innovative project is multi-disciplinary and brings a history of successful research collaboration. This resubmission was viewed as highly responsive to prior critiques. Overall, the panel was enthusiastic about this application and identified several strengths, including the strong preliminary data, the inclusion of conceptual frameworks to guide the study, the use of multiple data sources, pilot data supporting feasibility of the approach, the inclusion of families with limited English proficiency, and the assessment of multiple aspects of parental participation. The environment is seen as supportive. During the discussion, the panel also noted a few weaknesses in the approach, such as concerns that the small number of Hispanic participants may not provide meaningful insights, limited clarity for the race-based analyses, lack of attention to track longer-term outcomes, and concern about generalizability. The panel concluded that these weaknesses were minor and addressable. Following the discussion, the panel agreed that this was an impressive application from an exceptional group of investigators, and the findings are expected to have high impact on the field.

DESCRIPTION (provided by applicant): While some barriers and facilitators to parent presence and participation (PPP) in the neonatal intensive care unit (NICU) have been identified, comprehensive descriptions of factors influencing PPP and changes in these factors over time are lacking. Moreover, race- and socioeconomic-based disparities in PPP have been reported, but the underlying causes of these disparities are unknown. To increase health equity for preterm infants and their parents, the underlying factors influencing disparities in PPP must be identified. PPP is critical to improving clinical outcomes and neurodevelopment for preterm infants. The purpose of the proposed study is to comprehensively determine barriers and facilitators affecting PPP in the NICU and effects of PPP on infant outcomes. Using a non-experimental, longitudinal design, the following specific aims and subaims will be accomplished: (Aim 1) determine barriers and facilitators affecting PPP and changes in barriers and facilitators over time; (Subaim 1) predict barriers and facilitators of PPP based on sociodemographic characteristics; (Aim 2) determine the effect of PPP on infant clinical outcomes and neurodevelopment; (Subaim 2) determine the mediation effect of parent-infant responsiveness on the relationship between PPP and infant clinical outcomes and neurodevelopment; (Aim 3) determine the moderation effect of PPP on the relationship between infant stress exposure and infant clinical outcomes and neurodevelopment. Parents with an infant born less than 32 weeks gestational age will be enrolled. Parents will complete surveys throughout their infant's hospitalization to identify barriers and facilitators influencing PPP and to quantify their experiences of discrimination and parent-staff engagement in the NICU. PPP will be quantified by parent-report, electronic health record documentation, and NICU visitation logs. Infant clinical outcomes, including length of NICU stay and achievement of oral feeding competence, will be measured throughout hospitalization and at discharge. Neurodevelopment will be assessed at 3-months corrected age using the Test of Infant Motor Performance and 12-months corrected age using the Bayley Scales of Infant and Toddler Development, 4th Edition. Parent-infant responsiveness at discharge and 3-months corrected age will be determined as will the moderating effect of PPP on infant stress exposure throughout hospitalization. The study focuses on modifiable factors that may disparately affect parents from racially underrepresented groups or those lacking financial and social resources. By also considering demographic characteristics that may contribute to disparities, the study will provide data to support the

development of NICU interventions to equitably promote PPP, thus improving outcomes for preterm infants and their families. This proposal addresses the research priority area of the NICHD's Pregnancy and Perinatology Branch to advance the science of preterm birth and its cons equences while incorporating the NICHD's aspirational goal to enhance the healthy development of preterm infants and cross-cutting theme of addressing health disparities.

PUBLIC HEALTH RELEVANCE: The proposed project will determine the many influences on parent presence and participation in caregiving in the neonatal intensive care unit (NICU) and the importance of parent presence and participation for clinical and developmental outcomes of preterm infants. By studying these influences and how they change during an infant's NICU hospitalization, specific interventions can be developed to improve infant outcomes. The study will also provide information to better understand race- and socioeconomic-based disparities in parent presence and participation to increase health equity.

CRITIQUE 1

Significance: 2
Investigator(s): 1
Innovation: 4
Approach: 3
Environment: 1

Overall Impact: This proposal concerns parent presence and participation in care of their infant in the NICU. There is attention to health equity, particularly for preterm infants, for which there exist disparities in care and outcomes. This will be a non-experimental study to determine barriers and facilitators to parent presence, study effect of presence on outcomes, and the mediation of parent-infant responsiveness and outcomes such as neurodevelopment. Population is infants < 32 weeks gestation at birth. 3 month measurements will be assessed. The premise is important and sound that parental presence can impact later outcomes for this vulnerable population. This is an area that has gap in knowledge for which advancing this gap can promote interventions at the health system level. The investigative team is well suited to carry out this proposal which may have meaningful impact and draw further attention to this area of need, in particular to the gaps in health equity for this population.

1. Significance:

Strengths

- The premise that parent presence can impact outcomes is reasonably well established and also considered a family-centered approach to care.
- Disparities exist and are potential source of long-term inequities in healthcare for this population.
- Prior study on items like feeding readiness show benefit of parental participation. Skin to skin care is known to be of benefit.

Weaknesses

- Due to social factors, although knowledge may be gained, there may be constraints outside of the health system that may not be modifiable, such as policies and financial constraints.

2. Investigator(s):

Strengths

- The PI has clinical experience as NICU nurse and research experience on this topic.
- Co-investigators have appropriate experience in NICU care.
- There is experience in statistical design involving NICU patients and correlated data.
- Neonatologist Dr. Nelin has expertise in parental engagement intervention and research.

Weaknesses

- None noted by reviewer.

3. Innovation:

Strengths

- The longitudinal nature of the data analysis allows for time targeted factors to be known which may inform interventions.
- Qualitative portion allows for parental voice.
- A large range of data will be integrated into analysis, as well as multiple scales to study outcomes including parental well-being.

Weaknesses

- This is an observational study and so no intervention that may impact outcome is being proposed; although reasonable at this stage, it is unclear whether research will lead to clinical impact without some plan for designing an intervention.
- There may be opportunity for other data collection like biomarkers that could be thought of in the approach which may lead to increased knowledge and biological basis of the overall premise of benefit of parental presence.

4. Approach:

Strengths

- Statistical plan accounts for repeated measures considering longitudinal nature of data, as well as modeling to find key factors influencing the outcome of parental presence. Factors include system factors, social determinants, and parental factors including demographics and well-being.
- Plan to study Black families in particular as this is a group with disparate neonatal health outcomes.
- Inclusion of families with limited English proficiency is benefitting goal of health equity.
- A focus on health systems factors gives opportunity to plan for interventions that can have broad impact, vs those that may target individual families or clinicians.

Weaknesses

- The timeline for tracking longer-term outcomes is limited as the follow-up assessment is at 4 months corrected age and then at < 12 months, and the ability to distinguish meaningful long-term outcomes may not be possible or be as relevant that early in infancy.
- Data are obtained from the EMR, parent-report, and visitation logs. Validating some of these data points may not be available.

- More attention might be paid to time of day and visiting as there may be differential impact in this regard.

5. Environment:

Strengths

- Ohio State University College of Nursing is good environment to conduct this research.
- Nationwide Children’s Hospital NICU is appropriate clinical context.
- The Follow Up program facilitates data collection.

Weaknesses

- None noted by reviewer.

Study Timeline:

Strengths

- Appropriate.

Weaknesses

- None noted by reviewer.

Protections for Human Subjects:

Acceptable Risks and/or Adequate Protections

Data and Safety Monitoring Plan (Applicable for Clinical Trials Only):

Not Applicable (No Clinical Trials)

Inclusion Plans:

- Sex/Gender: Distribution justified scientifically
- Race/Ethnicity: Distribution justified scientifically
- For NIH-Defined Phase III trials, Plans for valid design and analysis: Not applicable
- Inclusion/Exclusion Based on Age: Distribution justified scientifically
- Appropriate.

Vertebrate Animals:

Not Applicable (No Vertebrate Animals)

Biohazards:

Not Applicable (No Biohazards)

Resubmission:

- The family burden has been reduced by reducing survey questions and points of contact.

Resource Sharing Plans:
Acceptable

Budget and Period of Support:
Recommend as Requested

CRITIQUE 2

Significance: 2
Investigator(s): 1
Innovation: 2
Approach: 3
Environment: 1

Overall Impact: This is a resubmission of an R01 proposal that uses a longitudinal cohort design to examine the association between parental presence and participation (PPP) in the NICU and infant outcomes over time and to identify multi-level barriers and facilitators of PPP. PPP has the potential to improve infant outcomes and reduce racial and socioeconomic inequities, but an understanding of key barriers and facilitators is needed to inform development of effective interventions. This study has the potential to significantly advance our understanding of these barriers and facilitators as well as the effects of PPP on infant clinical outcomes and neurodevelopment, and this has high potential impact. This proposal has been highly responsive to reviewer comments and key strengths include: a clear and appropriate conceptual framework guiding the proposal, a well-qualified study team, additional pilot data supporting feasibility of the approach, and a more comprehensive evaluation of parental participation. Weaknesses are minor and do not diminish the impact of the proposed work.

1. Significance:

Strengths

- Racial and socioeconomic disparities in infant outcomes are well-established. The identification of modifiable, multi-level barriers to PPP in the NICU setting has the potential to significantly improve infant outcomes and inform interventions that can more effectively address these inequities.
- The theoretical framework provides a well-developed approach to identifying salient multi-level factors and to distinguishing parent-level vs. system-level factors in particular.

Weaknesses

- None noted by reviewer.

2. Investigator(s):

Strengths

- Multidisciplinary team with experience in recruiting infants and parents for longitudinal observational studies and expertise with the proposed measures and outcomes. MPIs and co-Is with history of collaboration.

Weaknesses

- None noted by reviewer.

3. Innovation:

Strengths

- First study to prospectively examine factors that contribute to PPP in the NICU.
- Prior studies have focused on SSC, this study will examine multiple types of PPP, providing data on a more comprehensive PPP construct.
- Looking at measures of factors influencing PPP over time, given lengthy hospitalizations are common for study population, to develop time-targeted interventions.

Weaknesses

- None noted by reviewer.

4. Approach:

Strengths

- Strong conceptual framework to guide the multi-level evaluation of barriers and facilitators to PPP.
- Oversampling of Black families; feasible given demographics of the study sites.
- Survey burden has been decreased; pilot data supports feasibility of daily dairies with multiple routes of administration (paper/pen, electronically).
- Assessment of multiple aspects of parental participation (feeding, SSC, diaper changes, bathing).

Weaknesses

- Spanish-speaking participants will now be included (a strength) but anticipated enrollment data suggests few Hispanic patients. With such small numbers, it is not clear how meaningful insights will be generated by including Spanish-speaking participants. Language barriers are also not included in the PRBFQ, but may influence many of the barriers listed. (minor)
- Not clear how other races, including those who identify as multiracial will be treated in the race-based analyses for Sub-aim 1. (minor)

5. Environment:

Strengths

- Appropriate for the proposed study.

Weaknesses

- None noted by reviewer.

Study Timeline:

Strengths

- Appropriate.

Weaknesses

- None noted by reviewer.

Protections for Human Subjects:

Acceptable Risks and/or Adequate Protections

Data and Safety Monitoring Plan (Applicable for Clinical Trials Only):

Not Applicable (No Clinical Trials)

Inclusion Plans:

- Sex/Gender: Distribution justified scientifically
- Race/Ethnicity: Distribution justified scientifically
- For NIH-Defined Phase III trials, Plans for valid design and analysis: Not applicable
- Inclusion/Exclusion Based on Age: Distribution justified scientifically

Vertebrate Animals:

Not Applicable (No Vertebrate Animals)

Biohazards:

Not Applicable (No Biohazards)

Resubmission:

- Very responsive. Changes which have helped to strengthen this proposal include: clearer use of language to distinguish parent-level and system-level factors in the conceptual framework, decreased burden of surveys and new pilot data showing feasibiliy of the daily log, a more detailed analysis plan, and clarification around investigator roles.

Budget and Period of Support:

Recommend as Requested

CRITIQUE 3

Significance: 1
Investigator(s): 1
Innovation: 1
Approach: 2
Environment: 1

Overall Impact: The study is a resubmission focused on determining barriers and facilitators associated with presence and participation (PPP) in the NICU with a focus on health equity. The potential impact is high as it can help identify modifiable risk factors and inform interventions to improve health equity, with a focus on improving outcomes for racially marginalized populations. Overall strengths include longitudinal design, strong preliminary data, excellent selection of measures, use of

multiple data sources, and strong study team. Minor analytic concerns around approach for use of 2 vs 1 parent sources. Also, would want team to address study external validity.

1. Significance:

Strengths

- Addressing disparities around preterm birth is critically important area of research. Findings can inform much needed interventions with implications for infant outcomes and families.
- Strong theoretical model guiding research.

Weaknesses

- None noted by reviewer.

2. Investigator(s):

Strengths

- Strong team with complementary expertise and a range of backgrounds.
- Team has key expertise on recruitment of racial minorities in NICU settings.

Weaknesses

- None noted by reviewer.

3. Innovation:

Strengths

- Measurement of healthcare system factors related to PPP is novel and is critical to developing successful interventions.
- Use of multiple data sources allows for comprehensive evaluation of key factors.

Weaknesses

- None noted by reviewer.

4. Approach:

Strengths

- Strong preliminary data.
- Longitudinal study design which will include collection of longer-term outcomes.
- Multiple data sources are used for comprehensive evaluation of key factors.
- In response to reviewers comments from initial submission, team has reduced items and number of follow ups to limit participant burden.
- Thoughtful and comprehensive selection of key validated measures.

Weaknesses

- How are data collected from 2 parents vs 1 per family handled and is this a consideration in sub-group analyses by SES and race?

- Concerns about generalizability issues are not addressed. This may be particularly relevant to feelings of discrimination etc relative to characteristics of healthcare setting relative to diversity of patient composition and staffing.

5. Environment:

Strengths

- Ohio State University and Nationwide Children's Hospital provide the necessary resources and environment.

Weaknesses

- None noted by reviewer.

Study Timeline:

Strengths

- Reasonable as described. Some steps re: Spanish language translation and IRB are underway.

Weaknesses

- None noted by reviewer.

Protections for Human Subjects:

Acceptable Risks and/or Adequate Protections

Data and Safety Monitoring Plan (Applicable for Clinical Trials Only):

Not Applicable (No Clinical Trials)

Inclusion Plans:

- Sex/Gender: Distribution justified scientifically
- Race/Ethnicity: Distribution justified scientifically
- For NIH-Defined Phase III trials, Plans for valid design and analysis: Not applicable
- Inclusion/Exclusion Based on Age: Distribution justified scientifically

Vertebrate Animals:

Not Applicable (No Vertebrate Animals)

Biohazards:

Not Applicable (No Biohazards)

Resubmission:

- Concerns re: study burden and theoretical approaches addressed appropriately.

Budget and Period of Support:

Recommend as Requested

THE FOLLOWING SECTIONS WERE PREPARED BY THE SCIENTIFIC REVIEW OFFICER TO SUMMARIZE THE OUTCOME OF DISCUSSIONS OF THE REVIEW COMMITTEE, OR REVIEWERS' WRITTEN CRITIQUES, ON THE FOLLOWING ISSUES:

PROTECTION OF HUMAN SUBJECTS: ACCEPTABLE

INCLUSION OF WOMEN PLAN: ACCEPTABLE

INCLUSION OF MINORITIES PLAN: ACCEPTABLE

INCLUSION ACROSS THE LIFESPAN: ACCEPTABLE

COMMITTEE BUDGET RECOMMENDATIONS: The budget was recommended as requested.

Footnotes for 1R01HD113525-01A1; PI Name: NIST, MARLIESE D

NIH has modified its policy regarding the receipt of resubmissions (amended applications).See Guide Notice NOT-OD-18-197 at https://grants.nih.gov/grants/guide/notice-files/NOT-OD-18-197.html. The impact/priority score is calculated after discussion of an application by averaging the overall scores (1-9) given by all voting reviewers on the committee and multiplying by 10. The criterion scores are submitted prior to the meeting by the individual reviewers assigned to an application, and are not discussed specifically at the review meeting or calculated into the overall impact score. Some applications also receive a percentile ranking. For details on the review process, see http://grants.nih.gov/grants/peer_review_process.htm#scoring.
